# Different Features of Cholera in Malnourished and Non-Malnourished Children: Analysis of 20 Years of Surveillance Data from a Large Diarrheal Disease Hospital in Urban Bangladesh

**DOI:** 10.3390/children9020137

**Published:** 2022-01-20

**Authors:** Sharika Nuzhat, Md Iqbal Hossain, Nusrat Jahan Shaly, Rafiqul Islam, Soroar Hossain Khan, Abu Syed Golam Faruque, Pradip Kumar Bardhan, Azharul Islam Khan, Mohammod Jobayer Chisti, Tahmeed Ahmed

**Affiliations:** International Centre for Diarrhoeal Disease Research, Nutrition and Clinical Services Division, Bangladesh (icddr,b), Mohakhali, Dhaka 1212, Bangladesh; sharika.nuzhat@icddrb.org (S.N.); ihossain@icddrb.org (M.I.H.); nusrat.jahan@icddrb.org (N.J.S.); rafiqul@icddrb.org (R.I.); soroar@icddrb.org (S.H.K.); gfaruque@icddrb.org (A.S.G.F.); bardhan@icddrb.org (P.K.B.); azharul@icddrb.org (A.I.K.); tahmeed@icddrb.org (T.A.)

**Keywords:** cholera, malnutrition, dehydration, under-five children

## Abstract

Malnourished children are more prone to infectious diseases including severe diarrhea compared to non-malnourished children. However, data are scarce on differences in the presentation in such children. We aimed to identify clinical differentials among children with cholera with or without malnutrition. Data were extracted from the diarrheal disease surveillance system (DDSS) of Dhaka Hospital of International Centre for Diarrheal Disease Research, Bangladesh (icddr,b) from January 2001 to December 2020. Among children under five in DDSS, cholera positive (culture confirmed) malnourished children (WAZ, WL/HZ or L/HAZ ˂ −2) were considered as cases (*n* = 920) and children with cholera but non-malnourished (WAZ, WL/HZ or L/HAZ ≥−2.00 to ≤+2.00) were controls (*n* = 586). After adjusting for potential confounders such as maternal illiteracy, day labor fathers, maternal employment, slum dwelling, non-sanitary latrine use, use of untreated water, and history of cough, it was revealed that malnourished cholera children significantly more often presented in hospital during evening hours (6 p.m. to 12 mid-night) (*p* < 0.05), had illiterate fathers (*p* < 0.05), >24 h history of diarrheal duration (*p* < 0.05), dehydrating diarrhea (*p* < 0.05), and had longer hospitalization (*p* < 0.05). The study results underscore the importance of understanding of basic differences in the presentation of severity of cholera in malnourished children for prompt identification and subsequent management of these vulnerable children.

## 1. Introduction

Cholera is a leading public health concern globally, with an estimated 1.3–4.0 million cases occurring each year, worldwide [1]. A significant disease burden of cholera has been reported in young children [2,3]. In October 2017, the WHO and its partners under the auspices of the Global Task Force on Cholera Control (GTFCC) announced a target, Ending Cholera: A Global Roadmap to 2030 [4]. Malnourished children are at a higher risk of developing severe diarrhea with longer duration that is often associated with a fatal outcome [5]. The disease severity has been found to be associated with nutritional status, body size, and etiologic agents of diarrheal episodes [6,7]. Higher rates of malnutrition among pre-school children have been observed in Bangladesh [8]. A study in Brazil reported that the clinical presentations of early childhood severe diarrhea may vary because of diverse etiologies. Understanding of the differences in the presenting features of severe diarrhea especially in cholera that are associated with varying nutritional status of the young children is thus critically important for early identification and management these children. Hence, with an attempt to address the existing knowledge gap as well as to share research findings with policy makers for formulating better case management strategy we undertook this study to examine the clinical feature differentials among children infected with *Vibrio cholerae* who presented with or without malnutrition.

## 2. Materials and Methods

### 2.1. Ethical Statement

For this study, data were extracted from the electronic database of hospital-based diarrheal disease surveillance system (DDSS) of Dhaka Hospital of International Centre for Diarrheal Disease Research, Bangladesh (icddr,b). The DDSS has the approval from institutional review board (IRB) of icddr,b for data analysis. IRB of icddr,b comprises of Research Review Committee (RRC) and Ethical Review Committee (ERC). Protocol for DDSS was approved by IRB on 19 May 1992 (PR: 1992–011). ERC was also pleased with the voluntary participation, maintenance of rights of the participants and confidential handling of personal information by the hospital doctors and accepted this consenting procedure. At the time of enrollment into DDSS, verbal consent was obtained from the parents or the attending caregivers of each child following hospital policy. The verbal consent was recorded by a check mark in the questionnaire that was again assured by showing the mark to the parents or caregiver. DDSS is a routine ongoing surveillance in hospitals of icddr,b located in Dhaka, Bangladesh. At the time of consenting, parents or caregivers were assured of ‘any risk being no more than minimal risk’, ‘their participation is voluntary’, ‘their rights to withdraw from the study, and ‘the maintenance of strict confidentiality of disclosed information’. They were also informed about the use of collected data for analysis and using the results for improving patient care, conducting research, and also publication without disclosing the names or identities of their children. 

### 2.2. Study Population and Study Site

Dhaka Hospital of icddr,b provides care and free treatment to around 150,000 diarrheal disease patients each year and about 62% of them are children under five years of age. The DDSS systematically (from every 50th patient according to their hospital ID number) collects information including age, sex, socio-demographic characteristics, clinical features, and etiology of diarrhea. Parents or caregivers are interviewed by research assistants who collect demographic, socioeconomic, and clinical data. A physician documents the clinical findings including dehydration status. A fresh stool sample is collected and submitted to laboratory for microbiological evaluation. All relevant information is recorded into the electronic database as soon as possible. For the present study, analysis was limited to children under five who were cholera positive together with or without malnutrition and enrolled into the DDSS from January 2001 to December 2020. The nutritional status of these children was assessed at the time of discharge from the hospital. Weight was measured to the nearest 100 g using a digital scale and length/height was estimated using a locally manufactured length board with a precision of 0.1 cm. 

The nutritional status was expressed by Z-scores following WHO 2006 growth standards [9].

### 2.3. Study Design

A case control study design was followed. The study group (cases) comprised of malnourished diarrheal children with associated *Vibrio cholerae* infections and comparison group (control) was the cholera children without malnutrition who presented at the same time. 

### 2.4. Definition

Malnutrition was defined in children 0–59 months of age with any of the indices of malnutrition such as: weight for age z-score (WAZ) or height for age z-score (HAZ) or weight for height z-score (WAH) ˂ −2. Non-malnourished children had WAZ, HAZ, and WHZ ≥ −2.00 to ≤+2.00 [5]. Cholera case was defined by illness in under five children and infected with *V. cholerae* isolated by fecal culture.

### 2.5. Statistical Analysis

Data were analyzed using SPSS for windows (version 20; SPSS Inc., Chicago, IL, USA) and Epi Info version 7.0, USD, Stone Mountain, GA, USA. Differences in the proportion were compared by the Chi-square test. A probability value of <0.05 was considered to be statistically significant. Strength of association was determined by calculating odds ratios (OR) and their 95% confidence intervals (CI). Variables were selected after reviewing surveillance data record form. Logistic regression was performed to identify factors that were independently associated with malnourished cholera children after adjusting for potential confounding variables. Variables that were statically significant in the univariate analysis were included in the logistic regression model. Multicollinearity between independent variables was also checked before constructing logistic regression models and they demonstrated the variance < 3.0. 

## 3. Results

**Figure 1:**
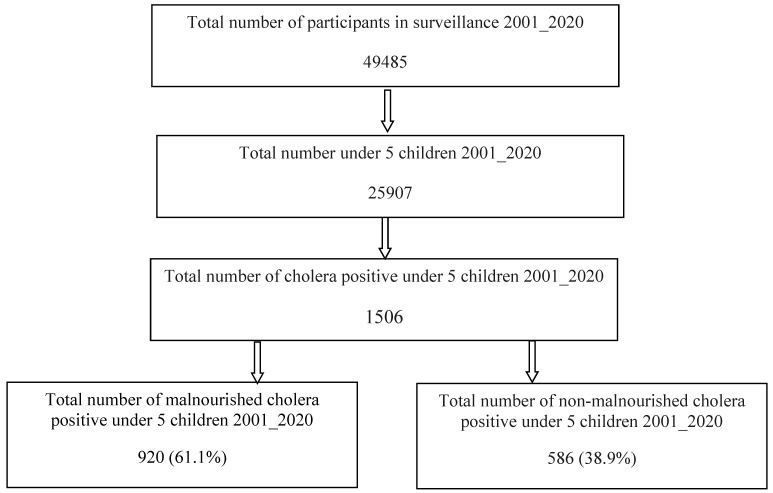
Showing the flow chart for selecting the cases and controls.

A total of 25,907 children under five were enrolled in the DDSS during the study period and 1506 children were found to have fecal culture proven cholera. Out of them, according to the eligibility criteria, 920 belonged to the study group (cases) while the rest constituted the comparison group (control). Figure 1 shows the flow chart for selecting the cases and controls.

Variables were selected from the data collect form of DDSS. Bi-variate analysis revealed that the cases more often had illiterate parents and lived in slum settlements compared to the controls. The cases compared to their counterpart commonly reported to the facility at evening hours (6 p.m.–12 mid-nights) with >24 h history of diarrheal illness, often had history of cough within the last seven days, and were observed to seek care often for to seek out care for dehydrating diarrhea. The cases often required longer hospitalization (>24 h) than the controls. Of mothers who presented to the facility with their child, 38% were illiterate and 72.1% of their children were malnourished. Among slum dwellers, 74.9% were malnourished. Proportions of cases and controls with regard to vomiting (90.2% vs. 88.6%), fever (2.6% vs. 2.7%) or abdominal pain (25.4% vs. 29.4%) were comparable. Mothers of malnourished cholera children mostly employed in any capacity (20.2% vs. 14.3%) and fathers were most often day laborers (8.4% vs. 5.6%) (Table 1).

Logistic regression analysis after adjusting for potential covariates such as maternal illiteracy, day labor fathers, maternal employment, slum dwelling, non-sanitary latrine uses of untreated water and history of cough, revealed that malnourished children under five years of age with cholera significantly more often had paternal illiteracy, reported to the hospital in evening hours, presented with dehydrating diarrhea, >24 h diarrheal illness, and had longer stay at hospital (Table 2). Among malnourished cholera children, 56.7% were wasted (WLZ/WHZ < −2), 65% were stunted (LAZ/HAZ < −2) and 83.7% were under weight (WAZ < −2) children.

## 4. Discussion

The present study observed different features of cholera in those cholera children with or without malnutrition. The most important significant observation is the higher risk of dehydrating diarrhea in malnourished cholera children than non-malnourished cholera children. Other important observations in malnourished cholera children than non-malnourished cholera children were: (i) frequent care seeking at evening-night hours, (ii) more than 24 h history of diarrhea and (iii) longer hospitalization. 

Suboptimum growth, according to anthropometric measures indicative of stunting, wasting, and low weight, has been shown to increase the risk of death from infectious diseases in childhood [10]. Stunting and underweight have the highest proportions of attributed child deaths, about 14% for both; wasting accounts for 12·6% (severe wasting 7·4%) of child deaths [11]. Therefore, based on the WHO Child Growth Standards we defined malnutrition of our study children.

Malnutrition is a cascade of biological, cultural and socio-economic interaction [12]. Data from demographic and health survey in Haiti identified an independent association between household food insecurity and cholera in common population though directionality of this association is unclear [13]. Food insecurity indirectly increases risk of cholera, by impacting behavior such as unsafe drinking water intake or eating unsafe food [13]. Despite improvements in the quality of life over decades, it is also documented that food insecurity, improper weaning, and infections, including intestinal parasitic infections, are still present in the community [14,15]. 

As expected in malnourished children, lack of immune responses, particularly the reduced secretory IgA levels in the gut mucosa along with hypochlorhydria or achlorhydria, made these study children more vulnerable to diarrhoeal illnesses with relatively lower inoculums. 

One possible explanation for more dehydrating diarrhea in malnourished cholera children is that these children are commonly slum dwellers with poor water-sanitation and hygienic practices that might have caused the ingestion of larger inoculums of *Vibrio cholerae* resulting in greater challenging dose of cholera toxin. Moreover, since malnourished children are likely to have an increased area of gut mucosal surface compared to their body weight than the non-malnourished children they are more vulnerable to higher purging rate and resultant greater stool output during diarrhea [6] In the case of malnourished children with cholera, slower turnover rate of gut mucosal cells, deficiencies of intestinal enzymes, micronutrients, and impaired immune responses with exposures to larger inoculums because of their dwelling in more contaminated environments in the slums might have caused more severe disease and delayed recovery, therefore, longer hospitalization.

Dewan et al. reported that children with associated *Vibrio cholerae* infections were significantly more severely underweight, stunted, and wasted. The study indicated that such associations may be due to an impaired gastric barrier, hypochlorhydria, and prolonged intestinal mucosal injury that are commonly observed in malnourished children [16]. A study in urban Bangladesh evaluated the role of common diarrheal pathogens and revealed that children with *Vibrio cholerae* infections were 5.5 times more likely to be associated with dehydrating diarrhea than in the case of children without this causative agent [17] and these children were significantly more malnourished. Similar to our findings, other researchers also revealed deterioration of the course of diarrhea in undernourished children [18,19]. Exerting greater emphasis on stool output, about five decades back, Palmer et al. indicated that higher inoculum size is likely to cause greater intestinal mucosal surface involvement causing higher stool volume per unit of time than a longer duration of diarrhea [18]. However, recent research reported that bacteriophase that are lytic for *V. cholerae* O1, O139 may modulate disease severity and duration of cholera outbreak [20]. On the basis of a study in the rural settings of Bangladesh, Black et al. reported that a child’s small body size because of young age and low nutritional status is more likely to exhibit more fluid loss (per kg body weight) during diarrhea and such children are more vulnerable to severe dehydration and death if not properly treated by the appropriate rehydration therapy [6]. Most studies that reported association between malnutrition and severity of diarrhea did not take into consideration the role of enteropathogens in causing severity of dehydration and longer duration of the episode [16,21,22]. Another study in Brazil reported that children with fever, vomiting or both would capture 75% of the children at risk of dehydrating diarrhea [23]. However, this study compared the inpatient cases versus outpatient controls without relating to the etiology and nutritional status. A study in Bangladesh among under 2 years old diarrheal children observed maximum sensitivity (77.5%) and specificity (91.2%) of predicting development of dehydration with the combination of vomiting, use of ORS at home, unhygienic maternal hands and residence more than 3 km away from a health facility [24]. In our study, particularly with cholera positive children, vomiting, fever or abdominal pain did not demonstrate any significant difference in cholera children with or without malnutrition. 

Victora et al. observed a strong association between low body weight of infants (regardless of age) and risk of higher dehydration. Low body weight was observed to be a superior determinant in comparison to the anthropometric indices for predicting dehydrating diarrhea in children reporting to the health facility. The study mentioned that children with low body weight are young, malnourished, or both. Children have a larger gut surface compared to their body size in addition to a greater purging rate due to diarrhea as compared to older children [21]. Another explanation of life-threatening dehydration among children is water that constitutes a greater proportion of children’s bodyweight. Young children use more water in a day for their higher metabolic rates, and the capability of their kidneys to conserve water is less compared to older children and adults [25]. Undernourished children with poor living environments are more susceptible to severe diarrhoeal disease and dehydration than healthy children [25]. Thus, malnourished cholera children during hospitalization require intensive treatment with adjunct appropriate antimicrobial and zinc therapy, careful assessments of dehydration at intervals, appropriate dietary intervention with closer follow-up. Malnourished children are more prevalent in families with low income and poor housing along with compromised water-sanitation and hygienic practices. These children should be targeted for health education at the household level along with support for continued breast feeding and initiation of rehydration therapy soon after the onset of diarrheal illnesses to prevent severity of disease with early referral of dehydrating children to appropriate facilities to avoid unnecessary death.

Our study observed that mothers of malnourished children were mostly working mothers compared to mothers of non-malnourished children, though we could not measure working hours of parents. Additionally, it was observed that fathers of malnourished children were often involved in low paid jobs/wage earnings like day laborer These malnourished cholera children were mostly from the urban slums and due to the nature and working hours of the father or mother or both they presented to the facility during evening hours or later. It was also well observed that lack of knowledge or impoverished condition might lead them to report later. In addition to education on nutrition in every level of management, it is also important to inform them on early reporting to the hospital during illness that may help to minimize morbidity, reduce hospital stay and financial loss. 

Another important observation from our study was that the malnourished cholera children reported a greater frequency of a history of cough within the last seven days, though in logistic regression analysis it was marginally significant. Cough is one of the key clinical features of respiratory tract infections. Malnourished children are prone to develop any infection [26] and the most common infections for malnourished children are gastrointestinal and respiratory infections [27,28]. The first line of defense mechanisms for these infections is the innate immunity, particularly the epithelial barriers and the mucosal immune response [29,30]. Malnourished children significantly suffer from compromised mucosal barriers of the gastrointestinal, respiratory and urogenital tracts.

The study may be replicated in other geographical and cultural settings to see if the same clinical features play a similar role in causing dehydrating diarrhea in malnourished cholera children. Cholera outbreaks in emergency settings, such as settlements for displaced population and in the aftermath of natural calamities, are now commonly encountered. In such situations, the treatment of children with severe acute malnutrition and cholera is difficult in terms of both competences of clinicians well as coordination of logistics. The findings of our study are likely to be very helpful in such situations emphasizing the critical need in keeping SAM children with cholera for a longer period of time in the diarrhea treatment center before their referral to a nutritional rehabilitation or outpatient treatment center. 

This study was conducted in an urban hospital and the vast majority of the patients represented a poor socio-economic background. Our study children had a higher degree of infection that required hospitalization because of severe illness and they represented a relatively small proportion of children while the vast majority of children with less severe disease received care at the household level and did not seek care from the present facility. Respondents were mothers who presented at the facility with their child; 38% had no formal schooling and 44.9% of their children were malnourished. Of those who presented from urban slums, 74.9% were malnourished. We could not assess the parent’s knowledge of malnutrition, diarrheal management, etc. One of the limitations of our study was a lack of data on fluid volume received during hospitalization and therefore did not have information regarding under hydration or over hydration. Thus, our study children might not be representing the greater population as well as geographical variations. There may have been limitations of anthropometric data that were subject to measurement error. However, our results are likely to generate several hypotheses.

Future studies could better describe the changes in the presenting features along with etiology specific changes in clinical features in malnourished children over time period. Along with the unbiased systematic collection of data, a larger sample size, high quality laboratory performance and use of probing techniques in interviewing of mothers or caregivers were thus the strengths of the study.

## Figures and Tables

**Table 1:** Socio-demographic and clinical findings of cholera children without or with malnutrition in Bangladesh (2001–2020).

Characteristics	Malnourished Cholera Children (*n* = 920) (%)	Non-malnourished Cholera Children (*n* = 586) (%)	OR	95% CI	*p* Value
Male	526 (57.2%)	329 (56.1%)	1.04	8.45–1.28	0.734
Illiterate mother	413 (44.9%)	160 (27.3%)	2.17	1.73–2.71	0.000
Illiterate father	424 (46.1%)	158 (27%)	2.32	1.85–2.90	0.000
Day labor father	77 (8.4%)	33 (5.6%)	1.53	1.00–2.33	0.059
Mother with any employment	186 (20.2%)	84 (14.3%)	1.51	1.14–2.01	0.005
Slum area	137 (14.9%)	46 (7.8%)	2.14	1.50–3.04	0.000
Non-sanitary Toilet	388 (42.2%)	170 (29%)	1.78	1.43–2.23	0.000
Use of untreated drinking water	654 (71.1%)	362 (61.8%)	1.52	1.22–1.89	0.000
Presence of vomiting	830 (90.2%)	519 (88.6%)	1.19	0.85–1.66	0.350
Presence of fever	24 (2.6%)	16 (2.7%)	0.95	(0.50–1.81)	0.983
Presence of abdominal pain	234 (25.4%)	172 (29.4%)	0.82	0.65–1.03	0.107
History of cough within last 7 days	376 (40.9%)	179 (30.5%)	1.57	1.26–1.96	0.000
History of measles	33 (3.6%)	31 (5.3%)	0.67	0.40–1.10	0.142
Diarrheal duration > 24 h	538 (58.5%)	292 (49.8%)	1.20	0.97–1.49	0.001
Dehydration (some/severe)	762 (82.8%)	423 (72.2%)	1.86	1.45–2.38	0.000
Watery stool	895 (97.3%)	572 (97.6%)	0.88	0.45–1.70	0.822
Use of IV fluid	461 (50.2%)	272 (46.9%)	1.14	0.92–1.40	0.238
Reporting time (2005–2020) (6.00 p.m. to 12.00 mid-night)	121/504 (24%)	65/411 (15.8%)	1.68	1.20–2.35	0.003
Length of stay > 24 h	527/902 (58.4%)	259/553 (46.8%)	1.59	1.29–1.97	0.000
Death	0/902 (0.0%)	1/552 (0.2%)	-	-	0.380

% denotes percentage of cholera positive malnourished and non-malnourished group until mentioned otherwise.

**Table 2:** Multivariable logistic regression analysis that showed the factors associated with cholera children with malnutrition in Bangladesh, 2001 to 2020.

Characteristics of Cholera Children	Adjusted OR	95% CI	*p* Value
Illiterate mother	1.14	0.79–1.64	0.498
Illiterate father	1.64	1.16–2.33	0.005
Day labor father	1.31	0.71–2.44	0.386
Mother with any employment	1.40	0.96–2.03	0.077
Slum dwelling	1.14	0.68–1.89	0.616
Non-sanitary toilet	1.25	0.87–1.79	0.219
Use of untreated drinking water	1.10	0.81–1.49	0.533
History of cough within last 7 days	1.36	1.00–1.86	0.052
Diarrhoeal duration > 24 h	1.51	1.14–2.00	0.004
Reporting time (6.00 p.m. to 12.00 mid night)	1.52	1.07–2.16	0.019
Dehydration (some/severe)	1.72	1.23–2.40	0.001
Length of stay > 24 h	1.39	1.05–1.85	0.023

## Data Availability

This data set contains some personal information of the study patients (such as name, admission date, month, area of residence). Our IRB has required that the personal information of the participants is not disclosed. Thus, the policy of our center (icddr,b) is that we should not make the availability of whole data set in the manuscript, the supplemental files, or a public repository. However, data related to this manuscript are available upon request and for researchers who meet the criteria for access to confidential data may contact with Armana Ahmed (armana@icddrb.org) to the Research Administration of icddr,b (http://www.icddrb.org/) accessed on 15 January 2022.
